# Human development, inequality, and their associations with brain structure across 29 countries

**DOI:** 10.1192/j.eurpsy.2025.10060

**Published:** 2025-07-16

**Authors:** Vicente Medel, Luz M. Alliende, Richard Bethlehem, Jakob Seidlitz, Grace Ringlein, Celso Arango, Aurina Arnatkevičiūtė, Laila Asmal, Mark Bellgrove, Vivek Benegal, Miquel Bernardo, Pablo Billeke, Jorge Bosch-Bayard, Rodrigo Bressan, Geraldo Busatto, Mariana Castro, Tiffany Chaim-Avancini, Monise Costanzi, Leticia Czepielewski, Paola Dazzan, Camilo de la Fuente-Sandoval, Covadonga M. Diaz-Caneja, Marta Di Forti, Ana Maria Diaz-Zuluaga, Stefan Du Plessis, Fabio Duran, Sol Fittipaldi, Alex Fornito, Nelson Freimer, Ary Gadelha, Clarissa Gama, Ranjini Garani, Clemente Garcia-Rizo, Cecilia Gonzalez Campo, Alfonso Gonzalez-Valderrama, Salvador Guinjoan, Bharath Holla, Agustin Ibañez, Daniza Ivanovic, Andrea Jackowski, Pablo Leon-Ortiz, Christine Lochner, Carlos Lopez-Jaramillo, Hilmar Luckhoff, Raffael Massuda, Philip McGuire, Jun Miyata, Romina Mizrahi, Robin Murray, Aysegul Ozerdem, Pedro Pan, Mara Parellada, Lebogang Phahladira, Juan P. Ramirez-Mahaluf, Ramiro Reckziegel, Tiago Reis Marques, Francisco Reyes-Madrigal, Annerine Roos, Pedro Rosa, Giovanni Salum, Freda Scheffler, Gunter Schumann, Mauricio Serpa, Dan J. Stein, Angeles Tepper, Jeggan Tiego, Tsukasa Ueno, Juan Undurraga, Eduardo A Undurraga, Pedro Valdes-Sosa, Isabel Valli, Mirta Villarreal, Toby T Winton-Brown, Nefize Yalin, Francisco Zamorano, Marcus Zanetti, cVEDA, Anderson M Winkler, Sara Evans-Lacko, Nicolas A. Crossley

**Affiliations:** 1 Latin American Brain Health Institute (BrainLat), Universidad Adolfo Ibáñez, Santiago, Chile; 2 Department of Psychology, Northwestern University, Evanston, IL, USA; 3 Autism Research Centre, Department of Psychiatry, University of Cambridge, Cambridge UK; 4 Department of Psychology, University of Cambridge, Cambridge, UK; 5 Department of Psychiatry, University of Pennsylvania, Philadelphia, PA, USA; 6 Department of Child and Adolescent Psychiatry and Behavioral Science, The Children’s Hospital of Philadelphia, Philadelphia, PA, USA; 7 Penn-Children’s Hospital of Philadelphia Lifespan Brain Institute, University of Pennsylvania, Philadelphia, PA, USA; 8 Department of Biostatistics, Johns Hopkins Bloomberg School of Public Health, Baltimore, MD USA; 9 Department of Child and Adolescent Psychiatry, Institute of Psychiatry and Mental Health, Hospital General Universitario Gregorio Marañón, Instituto de Investigación Sanitaria Gregorio Marañón (IISGM), Centro de Investigación Biomédica en Red Salud Mental (CIBERSAM), Instituto de Salud Carlos III, School of Medicine, Universidad Complutense, Madrid, Spain; 10 The Turner Institute for Brain and Mental Health, School of Psychological Sciences, Monash University, Melbourne, Australia; 11 Monash Biomedical Imaging, Monash University, Melbourne, Australia; 12 Department of Psychiatry, Faculty of Medicine and Health Sciences, Stellenbosch University, Cape Town, South Africa; 13 Centre for Addiction Medicine, National Institute of Mental Health and Neurosciences (NIMHANS), Bengaluru, Karnataka, India; 14 Barcelona Clinic Schizophrenia Unit, Hospital Clínic de Barcelona, Departament de Medicina, Institut de Neurociències (UBNeuro), Universitat de Barcelona (UB), Institut d’Investigacions Biomèdiques, August Pi i Sunyer (IDIBAPS), Centro de Investigación Biomédica en Red Salud Mental (CIBERSAM), Instituto de Salud Carlos III (ISCIII), Barcelona, Spain; 15 Laboratorio de Neurociencia Social y Neuromodulación, Centro de Investigación en Complejidad Social (neuroCICS), Facultad de Gobierno, Universidad del Desarrollo, Santiago, Chile; 16 McGill Centre for Integrative Neuroscience, Ludmer Centre for Neuroinformatics and Mental Health, Montreal Neurological Institute, Montreal, Quebec, Canada; 17 Applied Neurocognitive Psychology Lab. Carl von Ossietzky Universitaet Oldenburg, Oldenburg, Germany; 18 Interdisciplinary Laboratory in Clinical Neuroscience (LiNC), Department of Psychiatry, Federal University of São Paulo, São Paulo, Brazil; 19 Departamento e Instituto de Psiquiatria, Hospital das Clínicas da Faculdade de Medicina da Universidade de São Paulo, São Paulo, Brazil; 20 Grupo de Investigación en Neurociencias Aplicadas a las Alteraciones de la Conducta (INAAC), Fleni-CONICET Neurosciences Institute (INEU), Ciudad Autónoma de Buenos Aires, Argentina; 21 Department of Psychiatry and Mental Health, School of Medicine, University of Buenos Aires, Ciudad Autónoma de Buenos Aires, Argentina; 22 Consejo Nacional de Investigaciones Científicas y Técnicas (CONICET), Ciudad Autónoma de Buenos Aires, Argentina; 23 Laboratory of Psychiatric Neuroimaging (LIM-21), Departamento e Instituto de Psiquiatria, Hospital das Clinicas Faculdade de Medicina Universidade de Sao Paulo (HCFMUSP), Faculdade de Medicina, Universidade de São Paulo, São Paulo, Brazil; 24 Laboratory of Molecular Psychiatry, Centro de Pesquisa Clínica, Hospital de Clínicas de Porto Alegre, Porto Alegre, Brazil; 25 Programa de Pós-Graduação em Psicologia, Instituto Psicologia, Universidade Federal do Rio Grande do Sul (UFRGS), Porto Alegre, Brazil; 26 Department of Psychological Medicine, Institute of Psychiatry, Psychology and Neuroscience, King’s College London, London, UK; 27 Laboratory of Experimental Psychiatry, Direction of Research, Instituto Nacional de Neurología y Neurocirugía, Mexico City, Mexico; 28 Social, Genetic and Developmental Psychiatry Centre, Institute of Psychiatry, Psychology and Neuroscience, King’s College London, London SE5 8AF, UK; 29 National Institute for Health Research (NIHR) Maudsley Biomedical Research Centre, South London and Maudsley National Health Service (NHS) Foundation Trust, King’s College London, London SE5 8AZ, UK; 30 Department of Psychiatry, Faculty of Medicine, University of Antioquia, Medellín, Colombia; 31 Center for Neurobehavioral Genetics, Jane and Terry Semel Institute for Neuroscience and Human Behavior Los Angeles, University of California Los Angeles (UCLA), Los Angeles, USA; 32 South African Medical Research Council (SAMRC) Genomics of Brain Disorders Unit, Cape Town, South Africa; 33 Cognitive Neuroscience Center (CNC), Universidad de San Andres, Victoria, Ciudad Autónoma de Buenos Aires, Argentina; 34 Global Brain Health Institute (GBHI), Trinity College Dublin (TCD), Dublin, Ireland; 35 Global Brain Health Institute (GBHI), University of California San Francisco (UCSF), San Francisco, California, USA; 36 Department of Psychiatry, Universidade Federal do Rio Grande do Sul (UFRGS), Hospital de Clnicas de Porto Alegre, Porto Alegre, Brazil; 37 Integrated Program in Neuroscience, McGill University, Montreal, Quebec, Canada; 38 Early Intervention Program, Instituto Psiquiátrico Dr. J. Horwitz Barak, Santiago, Chile; 39 School of Medicine, Universidad Finis Terrae, Santiago, Chile; 40 Laureate Institute for Brain Research, Tulsa, OK, USA; 41 Department of Integrative Medicine, NIMHANS, Bengaluru, Karnataka, India; 42 Accelerator Program for Discovery in Brain disorders using Stem cells (ADBS), Department of Psychiatry, NIMHANS, Bengaluru, Karnataka, India; 43 Department of Psychiatry, Universidade Federal de São Paulo, São Paulo, Brazil; 44 Department of Education, Information and Communications Technology (ICT) and Learning, Østfold University College, Halden, Norway; 45 South African Medical Research Council (SA MRC) Unit on Risk and Resilience in Mental Disorders, Department of Psychiatry, Stellenbosch University, Stellenbosch, South Africa; 46 Department of Psychiatry, Universidade Federal do Paraná (UFPR), Curitiba, Brazil; 47 Department of Psychiatry, University of Oxford, Oxford, UK; 48 Oxford Centre for Integrative Neuroimaging (OxCIN), University of Oxford, Oxford, UK; 49 NIHR Oxford Health Biomedical Research Centre, Oxford, UK; 50 Oxford Health National Health Service (NHS) Foundation Trust, Oxford, UK; 51 Department of Psychiatry, Aichi Medical University, Aichi, Japan; 52 Department of Psychiatry, Graduate School of Medicine, Kyoto University, Kyoto, Japan; 53 Clinical and Translational Sciences Lab, McGill University, Douglas Mental Health University Institute, Montreal, QC, Canada; 54 Department of Psychiatry, McGill University, Montreal, Canada; 55 Department of Psychosis Studies, Institute of Psychiatry, Psychology and Neuroscience, King’s College, London, UK; 56 Department of Psychiatry and Psychology, Mayo Clinic, Rochester, USA; 57 National Institute of Developmental Psychiatry for Children and Adolescents (INPD), São Paulo, Brazil; 58 Department of Psychiatry, School of Medicine, Pontificia Universidad Católica de Chile, Santiago, Chile; 59 South African Medical Research Council (SA MRC) Unit on Risk and Resilience in Mental Disorders, Department of Psychiatry and Neuroscience Institute, University of Cape Town, Cape Town, South Africa; 60 Child Mind Institute, New York, NY, USA; 61 Department of Psychiatry and Mental Health, University of Cape Town, Cape Town, South Africa; 62 Neuroscience Institute, University of Cape Town, Cape Town, South Africa; 63 Centre for Population Neuroscience and Stratified Medicine (PONS), Institute for Science and Technology for Brain-inspired Intelligence, Fudan University, Shanghai, China; 64 PONS-Centre, Charité Mental Health, Dept of Psychiatry and Psychotherapy, Charité Campus Mitte, Berlin, Germany; 65 Integrated Clinical Education Center, Kyoto University Hospital, Kyoto, Japan; 66 Department of Neurology and Psychiatry, Faculty of Medicine, Clínica Alemana Universidad del Desarrollo, Vitacura, Santiago, Chile; 67 Escuela de Gobierno, Pontificia Universidad Católica de Chile, Santiago, Chile; 68 Research Center for Integrated Disaster Risk Management (CIGIDEN), Santiago, Chile; 69 Canadian Institute for Advanced Research (CIFAR) Azrieli Global Scholars Program, CIFAR, Toronto, Canada; 70 The Clinical Hospital of Chengdu Brain Science Institute, University of Electronic Science and Technology of China, Chengdu, China; 71 Centro de Neurociencias de Cuba, La Habana, Cuba; 72 FIDMAG Germanes Hospitalaries Research Foundation, Barcelona, Spain; 73 Department of Physics, Universidad de Buenos Aires, Ciudad Autónoma de Buenos Aires, Argentina; 74 Department of Neuroscience, Central Clinical School, Monash University, Melbourne, Victoria, Australia; 75 Department of Psychiatry, Alfred Health, Melbourne, Victoria, Australia; 76 Centre for Affective Disorders, Department of Psychological Medicine, Institute of Psychiatry, Psychology and Neuroscience, King’s College London, London, UK; 77 South London and Maudsley National Health Service (NHS) Foundation Trust, London, UK; 78 Unidad de Imágenes Cuantitativas Avanzadas, Departamento de Imágenes, Clínica Alemana de Santiago, Universidad del Desarrollo, Santiago, Chile; 79 Facultad de Ciencias para el Cuidado de la Salud, Campus Los Leones, Universidad San Sebastián, Santiago, Chile; 80 Hospital Sírio-Libanês, São Paulo, Brazil; 81 Department of Human Genetics, University of Texas Rio Grande Valley, Brownsville, USA; 82 Care Policy and Evaluation Centre, London School of Economics and Political Science, London, UK

**Keywords:** brain structure, inequality, meta-regression, MRI, social determinants of health

## Abstract

**Background:**

The macro-social and environmental conditions in which people live, such as the level of a country’s development or inequality, are associated with brain-related disorders. However, the relationship between these systemic environmental factors and the brain remains unclear. We aimed to determine the association between the level of development and inequality of a country and the brain structure of healthy adults.

**Methods:**

We conducted a cross-sectional study pooling brain imaging (T1-based) data from 145 magnetic resonance imaging (MRI) studies in 7,962 healthy adults (4,110 women) in 29 different countries. We used a meta-regression approach to relate the brain structure to the country’s level of development and inequality.

**Results:**

Higher human development was consistently associated with larger hippocampi and more expanded global cortical surface area, particularly in frontal areas. Increased inequality was most consistently associated with smaller hippocampal volume and thinner cortical thickness across the brain.

**Conclusions:**

Our results suggest that the macro-economic conditions of a country are reflected in its inhabitants’ brains and may explain the different incidence of brain disorders across the world. The observed variability of brain structure in health across countries should be considered when developing tools in the field of personalized or precision medicine that are intended to be used across the world.

## Introduction

The conditions in which people are born, grow, live, and age, also known as the social determinants of health [1], may increase the risk of developing brain-related disorders [2–4]. Furthermore, it has been proposed that these environmental factors are biologically embedded in the brain [5]. Studying the link between the brain and environmental risk factors using approaches such as neuroimaging is an established way to shed light on the neural underpinnings of the vulnerability to brain disorders in adverse conditions [6–8]. Arguably, this research has the potential to shape novel interventions and inform public health policies [9, 10].

The environment influences health at different interacting organizational levels, from the individual to the global [11, 12]. All levels within this complex system must be studied, since they cannot be reduced to a single (lower or higher) tier. The individual’s environment is shaped by their circumstances and personal history, which require assessments tailored to the uniqueness of their situation [13]. People also share common environmental exposures among different subgroups, communities, or even countries, which impact their outcomes [14]. Individuals may not be fully aware of their influence, and therefore, a study based on the individual experience of a specific exposure might miss its impact, as it has been widely discussed for racism [15]. Such group-level influences are seldom addressed in imaging research, with studies mostly focusing on individual exposures. For example, worse macro-economic conditions as those from low- and middle-income countries (LMIC), are associated with a higher proportion of dementia cases [16] and explain disparities in ageing processes [17, 18]. Despite some controversy [19], people from LMIC have a higher likelihood of depression [20, 21]. With regards to the potential brain mechanisms underlying these effects, previous studies have consistently found associations between poverty, hippocampal volume, and total cortical surface area [22–25].

Some properties of the social environment, such as exposure to inequality, are better defined for groups of people rather than individuals, which makes it difficult to study in a typical individual-based MRI study. Income inequality has been associated with worse health [26], including mental health [27]. The proposed mechanism of income inequality has been attributed to “social stress” related to relative status hierarchies rather than absolute wealth [28]. Social stress related to stigma has been associated with hippocampal volume decreases [29], and general stress with cortical thinning of frontoparietal regions [30, 31].

The macro-social and environmental factors that impact health have been mostly studied using epidemiological approaches based on large surveys or administrative data, which typically do not provide detailed biological information such as brain scans. Recently, we and others [9, 32] have shown that the macro-social organization of the state or country where people live is reflected in their brain structure [33]. Here we sought to advance our understanding of how the brain is impacted by the social determinants of health at the country-level. We therefore examined the associations between the brain structure of healthy adults with country-level indices of development and inequality across 29 different countries using a meta-regression approach. We used the United Nations’ Human Development Index as a measure of country-level development, a composite measure which includes economic activity (income), education (years of education), and health (lifespan) of the population [34]. To index inequality, we used the United Nations’ metric based on the distributions of income, education, and health. Our novel approach could be considered an ecological imaging approach, in which properties of groups of people are associated with their average brain structure. We hypothesized that human development across countries would be positively associated with total cortical area surface and hippocampal volume. Echoing stress-related changes reported in the literature, we hypothesized that exposure to inequality would be negatively correlated with hippocampal volumes and frontoparietal cortical thickness.

## Methods and materials

### Search strategy

Our main aim was to include MRI data from a wide range of countries as required by our method. We therefore included open access data reporting MRI images from healthy adults, collating several databases until November 2021 (Supplementary Figure S1). We were particularly interested in studies performed in under-represented countries and included data from collaborators across the world [32].

### Inclusion criteria

We included samples approved by the local ethics committee that reported T1-weighted MRIs from at least 15 adults reported as healthy aged 18–40 years (inclusive). Images were acquired on 1.5 T and 3 T MRI scanners. We excluded samples obtained from 7 T MRI scanners because they require adjustments to the data processing pipeline, rendering their results less comparable [35]. Additionally, their prevalence in countries with high human development levels could introduce significant bias.

### Individual-level information extracted

At the individual level, we collected age and gender for every participant. Considering that pseudo-anonymized data acquired for diverse purposes and released to the public have little other information, we were not able to extract important aspects such as the socioeconomic characteristics (income or level of education) of the participants.

### Pre-processing of imaging data

All imaging data were processed using FreeSurfer’s cortical reconstruction pipeline recon-all (see Supplementary Table S1 for details of the specific version used). We examined associations with total intracranial volume (eTIV), hippocampal volume, and morphometric properties of the cortex, including thickness and surface area from the two hemispheres and the 68 cortical regions of interest (ROI) of the Desikan–Killiany atlas [36]. Quality control was based on an initial visual inspection, which was in some samples performed locally by collaborating groups (Supplementary Table S1) or by two reviewers. It was then followed up by an automatic quality control procedure in which participants were excluded if any of the morphometric properties including any ROIs, either in thickness or surface, were outliers in their study sample as defined by Tukey’s fence:
[1]

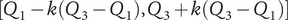

where *Q* refers to the respective quartile and using a *k* = 3 so that values that are “far out” were identified [37].

Considering the consistent difference observed between genders in head size [38], we also examined a scatterplot of the difference between women and men in intracranial volume as quality control of data labeling, excluding one dataset that was an extreme outlier.

### Country-level measures of development and inequality

Country-level characteristics were obtained from the United Nations Development Program from data published for the year 2019. These included the Human Development Index (HDI) and an inequality factor. The human development index was created to emphasize that people and their capabilities should be the criteria to assess the development of a country, and not only the national income. It is built using the geometric mean from indices in three key dimensions of human development: a long and healthy life (measured using the life expectancy at birth), access to knowledge (using the mean between the expected years of schooling and average years of schooling) and a decent standard of living (gross national income per capita) [39, 40]. It first defines minimum and maximum values for each dimension, and a normalized value is calculated using the following formula:
[2]

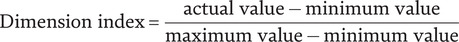

The coefficient of human inequality draws on the Atkinson’s inequality measures [41], which are widely used in the Social Sciences. Alongside a measure of distribution, it also includes an aversion parameter, which is adjusted to reflect society’s sensitivity to inequality. For the HDI, the United Nations sets this aversion *ε* parameter to 1 (i.e., equal weight is given to everyone’s welfare in society), leading to:
[3]

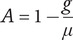

where *g* is the geometric mean of the distribution and *μ* is the arithmetic mean. These inequality indices *A* are measured for each dimension, and pooled using their arithmetic mean. Since inequality is negatively associated with HDI (a correlation of −0.9 (*P* < 0.0001) across all countries for which the indices are reported), we regressed out the HDI. In other words, we are looking at brain associations with varying degrees of inequality at the same level of development. Most previous health studies examining inequality refer to income inequality. In this dataset, the multidimensional inequality index is highly correlated with income inequality (correlation of 0.86, *P* < 0.0001), so we could not examine if associations were driven by specific dimensions.

### Meta-regression analyses

We performed meta-regression analyses examining the associations between HDI and the brain, and separately between inequality and the brain. These analyses were performed on morphometric properties that in previous studies, at the individual level, have been most consistently associated with poverty and inequality: the eTIV, the cortex, and the hippocampi [24, 29]. For the cortex analyses, we focused on cortical thickness and surface area from the two hemispheres, and performed a regional analysis of the 68 regions of interest from the Desikan–Killiany atlas. For these latter analyses addressing localized differences in subregions within hemispheres, we corrected results for multiple testing using false-discovery-rate (*fdr*). The choice of cortical thickness and surface area over a combined metric such as volume was based on their differential genetic control and environmental influence [32, 42]. We used a random-effects model with weights based on the inverse of the variance of the imaging metric examined, modeling the between-study variance using the Paule and Mandel estimator [43]. To ensure that differences were not driven by sex or age differences, we included the mean age and the proportion of men as extra regressors.

### Examining the reliability of the results

Brain differences between countries could be due to differences in the scanner, or otherwise differences in the genetic or ethnic background of the population studied. We performed a jackknife analysis to examine if results were driven by a single site (leave-one- sample-out approach) and excluding all studies from a country (leave-one-country-out approach). We also performed analyses excluding all Western nations here defined as European countries, Canada, USA, and Australia.

To quantify the potential impact of data acquired with MRI scanners with different magnetic fields, namely 3 T and 1.5 T, we conducted sensitivity analyses incorporating the use of 1.5 T MRI scanner as an additional dummy regressor in our meta-regression analyses.

Countries and their populations undergo changes in time, including shifts in their level of development and inequality. To examine the potential impact of these changes, we used the HDI and inequality (adjusted) indices in the model corresponding to the year in which the images were obtained. Since open-access data do not have this information, we imputed it assuming it was three years prior to the publication date of the article associated with the dataset, when such a publication was available (see Supplementary Table S1). Note that inequality data for the Human Development Index was only published after 2010 by the United Nations, which restricts the scope of this analysis.

### Reporting effect sizes

**Figure 1.**
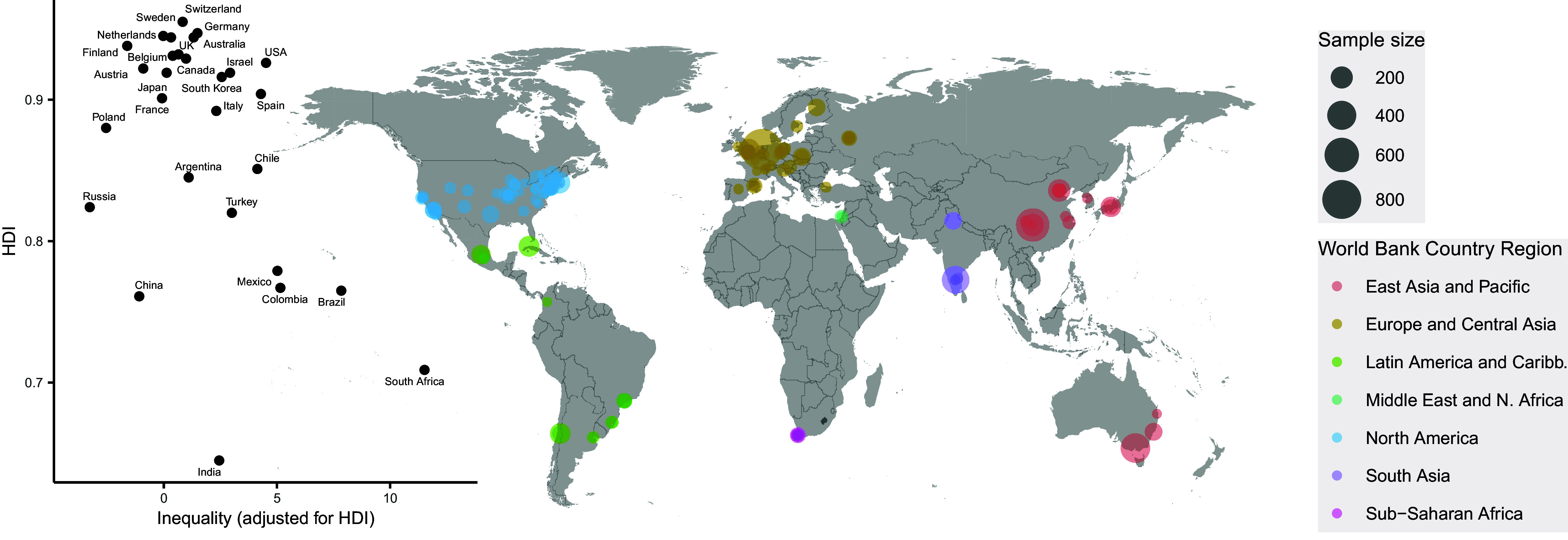
Geographical location of the samples included, their size, and characteristics of their countries. Interval highlighted in the graph (0.1 points for HDI and 5 points for inequality) are the intervals used to express effect sizes. Values of HDI and Inequality (adjusted) for each country included are also reported in Supplementary Table S2.

To facilitate the interpretation of results, beyond betas that may be difficult to understand in isolation, we report findings related to percent changes of brain structure using the average brain of the whole sample. We describe associations related to a 0.1 change in HDI and 5 points in the Inequality index adjusted for HDI (see Figure 1A to get an idea of this effect size).

Analyses were performed in R (4.3.1) using the Metafor package [44].

## Results

We included 145 samples of MRI images from 29 different countries from all groups of regions in the world (Figure 1; Supplementary Table S2). The total number of participants was 7,962, including 3,852 men and 4,110 women. Thirty-five percent of participants lived in a low-and-middle-income country (LMIC). The median average age of participants across samples was 24.2 years (range 18.3 to 31.7). Nearly 80% of the datasets were likely acquired after 2010 (Supplementary Table S1). Supplementary Figure S2 plots the changes in the development and inequality indices in those countries with datasets acquired in more than one year.

### Brain associations with human development

**Figure 2.**
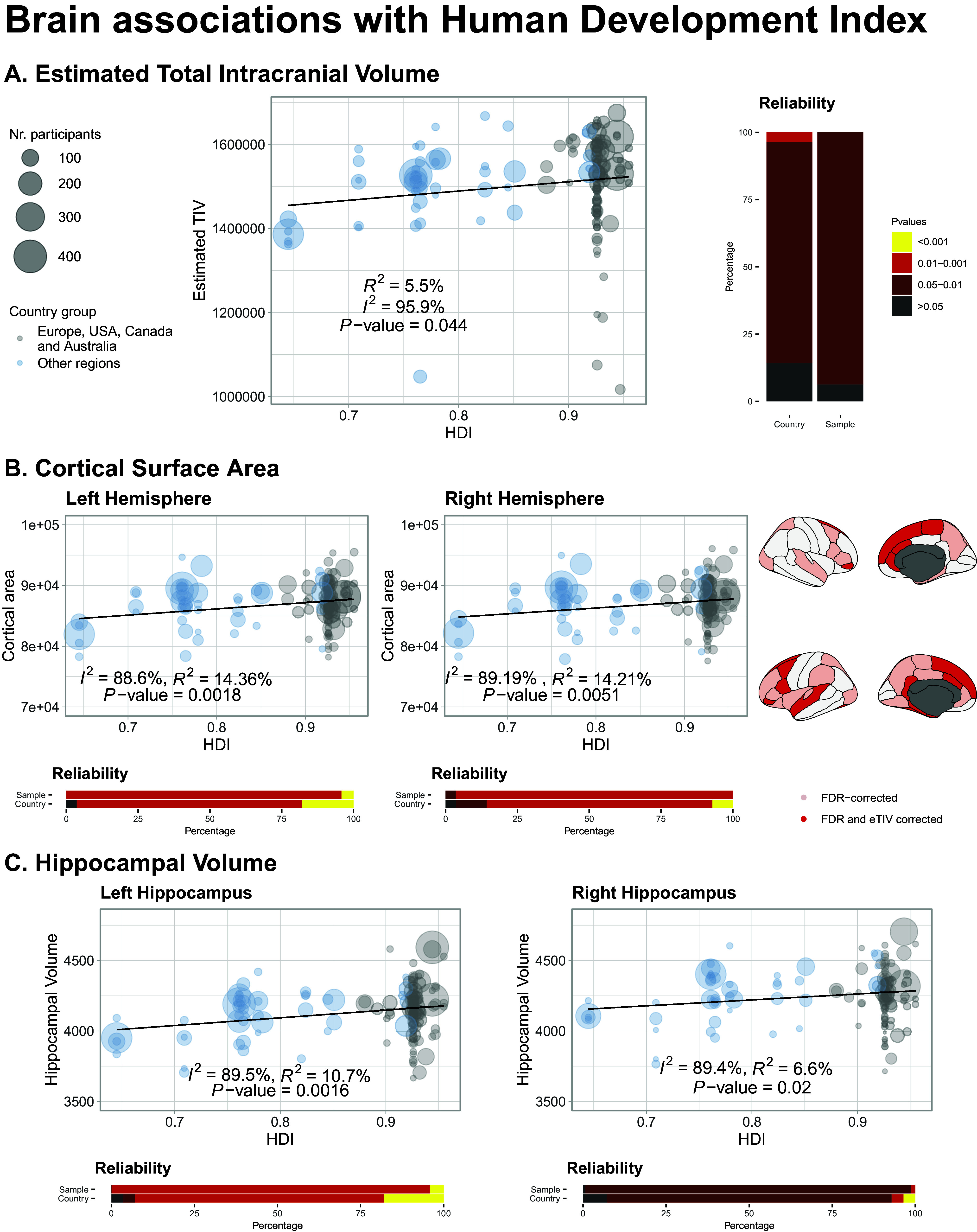
Brain associations with the Human Development Index. (A) Associations with estimated total intracranial volume. Reliability graph describes jack-knife analyses leaving-one- sample-out and leave-one-country-out. (B) Associations with hemispheric and regional surface. (C) Associations with hippocampal volume.

The country-level HDI was associated with total brain volume (eTIV or estimated total intracranial volume), with an increase of 0.1 points in the HDI associated with an increase of 1.57% (95% CI 0.07 to 3.06%, *P* = 0.041 and *R* ^2^ = 5.92%) (Figure 2A). Jackknife analyses showed that this result was significant 93.8% of the time when a study was excluded, and 85.7% when a country was excluded (Figure 2A). Subgroup analysis of non-Western countries was also significant, but the association was not present when considering Western countries.

The cortical surface area estimates for both hemispheres were positively correlated with HDI [1.14% increases (95% CI 0.34–1.94) for 0.1 change in HDI in the right hemisphere, *P* = 0.005; 1.22% (95% CI 0.45–1.99) in the left, *P* = 0.0018] (Figure 2B). These results were robust to leave-one-study-out, or one country out. They were also significant when controlling for the average total intracranial volume of each sample. The results remained unchanged for the left hemisphere only when considering non-Western countries, and they were not significant for Western countries only. Regional analyses corrected for *fdr* showed that the relationship of cortical surface area with both indices was widespread across the cortex but was particularly concentrated in frontal regions when accounting for the total intracranial volume (Figure 2B).

Hemispheric cortical thickness was not associated with HDI (left *P* = 0.86; right *P* = 0.88). Analyses on brain sub-regions showed a significant association between thickness and HDI in both right and left post-central gyri.

Hippocampal volume was positively associated with HDI in the left (1.36% increase per 0.1 change in HDI, 95% CI 0.52–2.21, *P* < 0.002) and right hemisphere (0.99%, 95% CI 0.14–1.84, *P* = 0.022) (Figure 2C). Results were robust to jackknife analyses as shown in Figure 2C. These associations were still significant when correcting for total brain volume for the left, but not the right, hippocampus. It was also present for studies including only non-Western countries, but not for Western countries.

Table 1A Provides a summary of all the findings related to HDI.

**Table 1.** Summary of main results

A. HDI																		
	Main analysis				Controlling for eTIV				Studies in Western countries				Studies in non-Western countries					
	Effect size*	95% CI		*P*-value	Effect size*	95% CI		*P*-value	Effect size*	95% CI		*P*-value	Effect size*	95% CI		*P*-value	Leave-one- sample out (%)	Leave-one country out (%)
eTIV	1.57	0.07	3.06	0.041	*NA*	*NA*	*NA*	*NA*	−6.11	−18.17	5.96	0.3211	4.52	1.80	7.23	0.0011	93.75	85.71
Right cort. Surface area	1.14	0.34	1.94	0.0051	1.07	0.12	2.02	0.0274	2.20	−3.45	7.84	0.4456	1.87	0.00	3.75	0.0503	100.00	96.43
Left cort. surface area	1.22	0.45	1.99	0.0018	0.93	0.21	1.66	0.0119	2.18	−3.22	7.58	0.4282	1.84	0.04	3.64	0.0450	100.00	96.43
Right cort. thickness	−2.17	−31.21	26.86	0.8834	*NA*	*NA*	*NA*	*NA*	*NA*	*NA*	*NA*	*NA*	*NA*	*NA*	*NA*	*NA*	*NA*	*NA*
Left cort. thickness	2.65	−26.84	32.15	0.8599	*NA*	*NA*	*NA*	*NA*	*NA*	*NA*	*NA*	*NA*	*NA*	*NA*	*NA*	*NA*	*NA*	*NA*
Right hippocampus	0.99	0.14	1.84	0.0220	0.78	−0.26	1.81	0.1433	0.66	−6.02	7.34	0.8463	3.33	1.73	4.92	0.0000	100.00	96.43
Left hippocampus	1.36	0.52	2.21	0.0016	1.31	0.27	2.35	0.0131	2.10	−4.84	9.04	0.5531	2.75	1.24	4.26	0.0004	100.00	96.43
									* = Effect size expressed in % increase associated with 0.1 increase in HDI									

B. Inequality (adjusted for HDI)																		
	Main analysis				Controlling for eTIV				Studies in Western countries				Studies in non-Western countries					
	Effect size#	95% CI		*P*-value	Effect size#	95% CI		*P*-value	Effect size#	95% CI		*P*-value	Effect size#	95% CI		*P*-value	Leave-one- sample out (%)	Leave-one country out (%)
eTIV	−2.10	−4.13	−0.06	0.0437	*NA*	*NA*	*NA*	*NA*	−2.12	−5.96	1.72	0.2785	−1.80	−4.29	0.68	0.1548	86.11	60.71
Right cort. surface area	−1.44	−2.55	−0.33	0.0109	−1.05	−2.10	0.00	0.0491	−1.85	−3.61	−0.08	0.0407	−1.34	−2.97	0.28	0.1048	100.00	92.86
Left cort. surface area	−1.37	−2.44	−0.30	0.0120	−0.98	−1.99	0.02	0.0556	−1.82	−3.50	−0.13	0.0343	−1.29	−2.85	0.27	0.1047	100.00	92.86
Right cort. thickness	−1.22	−1.98	−0.45	0.0018	*NA*	*NA*	*NA*	*NA*	−1.41	−2.99	0.18	0.0821	−1.01	−1.73	−0.30	0.0056	100.00	100.00
Left cort. thickness	−1.24	−2.01	−0.46	0.0017	*NA*	*NA*	*NA*	*NA*	−1.48	−3.09	0.12	0.0695	−1.00	−1.72	−0.27	0.0068	100.00	100.00
Right hippocampus	−1.98	−3.13	−0.84	0.0007	−1.41	−2.46	−0.37	0.0080	−0.47	−2.58	1.64	0.6636	−2.82	−4.17	−1.48	0.0000	100.00	100.00
Left hippocampus	−1.78	−2.90	−0.65	0.0020	−1.29	−2.35	−0.23	0.0175	−1.15	−3.34	1.03	0.3009	−2.35	−3.64	−1.05	0.0004	100.00	96.43
									# = effect size expressed in % decrease associated with 5 points increase in inequality									

### - Brain associations with inequality

We then examined the associations between brain structure and inequality (HDI-adjusted). We report effect sizes in percentage changes of the average structural brain associated to changes of 5 points in the Inequality index (see Figure 1A).

**Figure 3.**
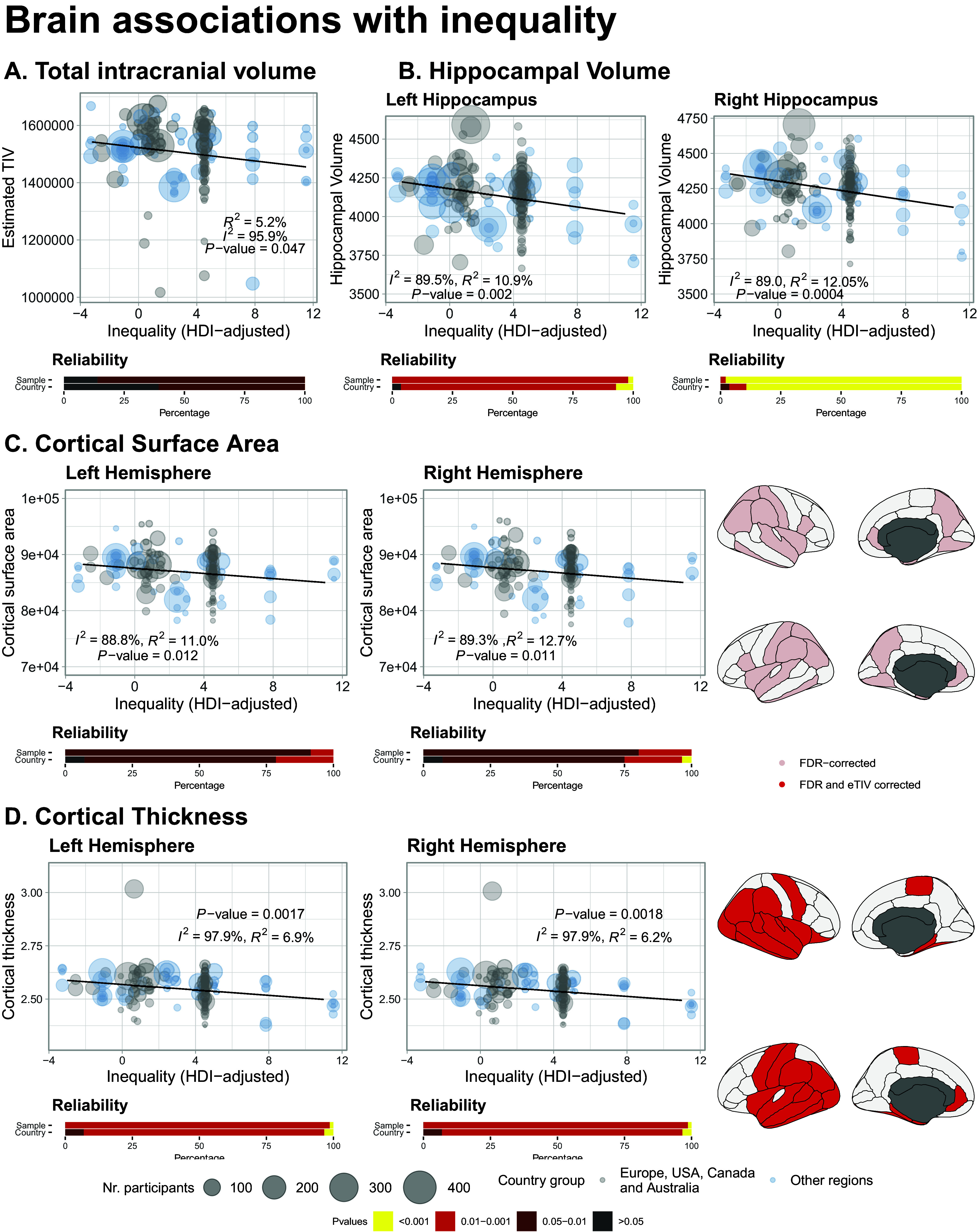
Brain associations with HDI-adjusted inequality. Associations with (A) estimated total intracranial volume, (B) hemispheric cortical surface area, as well as regional associations, (C) hemispheric and regional cortical thickness, and (D) hippocampal volume. In all panels heat maps represent leave-one-sample-out analyses and leave-one-country-out analyses (excluding all studies of a particular country). Note that no surface area results were significant after controlling for total intracranial volume.

Total intracranial volume was negatively associated with inequality (decreases of 2.1% associated with increases of 5 points in inequality, 95% CI −4.1 to −0.06%, *P* = 0.044) (Figure 3A). However, this result was reproduced in 86% of the leave-one-sample-out analyses, and 60.7% of the leave-one-country-out analyses. It was also not replicated in the analyses including only non-Western countries’ samples or Western countries’ samples.

There was a significant negative association with brain cortical hemispheric surface (right decreases of −1.44% for every 5-point increases, 95% CI −2.55 to −0.33, *P* = 0.011; left decreases of −1.37%, 95% CI −2.44 to −0.3, *P* = 0.012) (Figure 3B). Results were stable when performing leave-one-sample-out (100% in both hemispheres) and leave-one-country-out (96% in both hemispheres). When controlling for total intracranial volume, results were significant on the right but not left side. Results were significant when looking only at Western samples, with no significant associations in non-Western samples. This association was significant at the regional *fdr*-corrected level in areas such as bilateral entorhinal cortex and inferior temporal area; left superior temporal, temporal pole, supramarginal gyrus, and rostral anterior cingulate; and right insula and inferior parietal (*P_fdr_* < 0.05; Figure 3A). When controlling for total intracranial volume, none of these regional results were significant.

We also found a significant negative association between inequality and the right hemisphere mean cortical thickness (decreases of 1.22% per 5 points increase, 95% CI −1.98 to −0.45, *P*-value = 0.0018), as well as in the left hemisphere (decreases of 1.24% per 5 points increase, 95% CI −2.01 to −0.46, *P*-value = 0.0017; Figure 3B). These results were consistent in the leave-one-sample-out and leave-one-country-out analyses. It was also observed in samples including non-Western countries only, but not for Western countries. As shown in Figure 3B, this relationship was widespread in the brain, particularly in temporal and parietal regions.

Bilateral hippocampal volume was also associated with inequality (left hippocampus, decrease of 1.78% per 5 points increase, 95% CI −2.9 to −0.65, *P* = 0.002; right hippocampus, decrease of 1.98% per 5 points increase, 95% CI −3.13 to −0.84, *P* = 0.0007). This result was significant when controlling for total intracranial volume. It was robust to leave-one-sample-out (100% significant bilateral), and leave-one-country-out (96% significant on the left, 100% on the right). It was also present bilaterally in samples from non-Western countries, but not in Western samples.

Table 1B Provides a summary of all the findings related to inequality.

### - Sensitivity analyses

The use of 1.5 T MRI scanner instead of 3 T had no significant effect in the main analyses, as shown in Supplementary Figure S3.

Modeling the level of development and inequality of a country according to the imputed year in which MRI images were obtained did not change substantially the main results (Supplementary Figure S4). Some results were no longer significant for the inequality analyses, particularly hippocampal volume or right hemispheric thickness, which could be related to the lower power of the inequality analysis.

## Discussion

Our study provides further evidence of how macro-social and environmental factors are associated with brain structure in healthy adults. We found that higher country-level development was consistently and positively associated with hippocampal volume bilaterally and cortical surface area, even when controlling for total intracranial volume. Inequality on the other hand, was most consistently associated with thinner cortical thickness across the brain and smaller hippocampi volume, and less consistently with lower total intracranial volume and surface area, particularly in temporal and parietal surfaces.

These results could be compared to the many studies that have examined the effects of low socioeconomic status on the developing brain [22, 24, 25]. The country-level results echo similar findings from individuals within a community: higher levels of development are consistently associated with larger cortical surface area and larger hippocampi. This similarity is expected, given that the method used involves the average brain values across communities with different levels of development. Nonetheless, the consistency of these findings, combined with our robustness analyses, help to validate our novel approach. Our findings related to total brain size can also be read alongside the observed increases of head circumferences in children across the world in the last decades, which mirrors the increasing country development [45, 46]. The fact that better conditions lead to larger head circumferences in the next generations provides us with some clues about the causal direction of our observed association. More importantly, if we were to consider some of these brain morphometrics as integral part of brain health, they highlight that our indicators of worse brain health in some populations can be improved.

A similar expansion of brain area surface was seen in adults who participated in a randomized controlled trial of an educational intervention during childhood [47]. Therefore, one of the potential underlying mechanisms might be the greater availability of early educational opportunities in more developed countries. A larger head size after controlling for sex differences, often indicative of larger total intracranial volumes in healthy people, is also associated with lower dementia risks [48]. Considering the high burden of dementia in LMIC, smaller average brain size could be one aspect of a population-level marker of decreased brain reserve [49].

Lower human development is defined by the presence of worse material conditions, education and health. The different measures used to build the human development index are highly correlated across countries. It is therefore difficult to disentangle between the effects of material conditions, education or health with our neuroimaging ecological approach. At the same time, these dimensions are also associated with other environmental conditions that could affect brain health and development, such as poor nutrition, pollution or exposure to violence. Our current approach could not address their impact either. Future studies could examine communities where these dimensions and associated factors are less correlated, using natural or quasi-experimental designs that exploit external shocks, policy changes, or natural variations creating plausibly exogenous differences in exposure. For example, examining the impact of increasing the length of compulsory education, the implementation of direct cash transfers to improve material conditions, or the introduction of pollution-control technologies.

The novel ecological imaging approach adopted in this study allowed the examination of brain associations with inequality, which is difficult to address in an individual-based imaging study. We found that country-level inequality was robustly associated with lower hippocampal volume. This result is in line with animal models of social defeat and its impact on the hippocampus [50], as well as associations of smaller hippocampal volume in people who belong to a minority group [29]. We also found consistent associations with a thinner global cortical thickness, particularly in temporo-parietal regions. It remains unclear whether these associations could be attributed to a social stress mechanism, and further studies will have to examine further these suggestive findings. As has been proposed for the relationship between mortality and inequality [51], it is plausible that some of these findings are due to the use of aggregate data (ecological fallacy) in morphological characteristics that are solely influenced by development. This is particularly the case for our hippocampal results, where the observed association with development is unlikely to follow a simple linear association. It is less likely for thickness since this morphometric property was not associated with development.

The observed variability of brain structure in different environments echoes warnings from epidemiological science highlighting the existence of risk factors that affect populations and individuals [12]. In this context, case–control studies within a population might fail to elicit the population-level risks, which we have shown occurs in the typical case–control imaging studies in psychiatry [52]. Our results are also relevant for the field of personalized or precision medicine and psychiatry [53]. There are clear advantages of creating normative models of brain development for the definition of pathological states [54, 55]. However, while there is a recognized effort to include samples from diverse world regions to create those brain charts, particularly considering possible ethnic variations in brain shape [56, 57], current approaches have modelled site differences using age and sex and assumed that the rest of the variance is due to the inter-scanner difference. Little attention has been paid to the need to consider brain development unaffected by socio-environmental conditions when defining standards, as was done by the World Health Organization when creating their growth curves [58]. As we show here, brain metrics also reflect the population-level exposure to risk factors such as lower development. For machine-learning approaches, we should be careful to prevent the introduction of a representation bias when building these algorithms in a limited number of high-income countries [59]. Such a bias would unintendedly perpetuate existing health inequalities across the world. The results shown highlight the importance of integrating neuroscience with global mental health [60].

Our study relied on finding differences across sites and countries. We did not apply any harmonization procedure accounting for differences in acquisition across sites, as is now commonly done [61]. Controlling for scanner variability would have potentially masked site-specific differences, which were central to our study. Inter-site variability would have little impact on our findings as long as it was not correlated with HDI. As our sensitivity analyses showed, using a 1.5 T MRI scanner instead of a 3 T had a small impact on the results.

We should mention some limitations of this study. Previous studies have suggested that international differences in brain structure could be related to cultural factors shaping the brain [62] or genetic background [63], although the evidence suggest their effect is restricted to specific brain regions and are not associated with a global effect as we found [64]. Our confirmatory analyses on Western and non-Western countries aimed to explore this possibility. Our findings do not rule out the influence of cultural or genetic factors on brain structure, since much of this variance is independent from levels of development. For the correlated variance, particularly relating a possible impact of genetics on development and brain structure, we argue that the previously mentioned temporal trends within populations, linking larger head size with advances in development [45, 46], challenge this interpretation. We included only adults, a life stage where the brain is less influenced by development or aging processes. However, this choice limited our ability to study how macro-economic conditions affect these processes, such as identifying potential critical periods. Additionally, our results were not always replicated in subgroup analyses, especially in Western-only samples. This is likely due to the lower variance existing in HDI and inequality metrics in those countries, which reduced statistical power.

A further important point is that our study is based on samples that may not be representative of their broader country population, and we lacked individual-level data to address this limitation. Populations within countries are diverse, and imaging studies, even those using epidemiological sampling [65], often do not fully represent all communities. Despite these limitations, our study emphasizes the importance of considering the conditions in which people lived when data was collected, even using a broad classification such as country, when interpreting their findings [66]. We hope it will also encourage imaging researchers to provide information about the socioeconomic context in which their studies were performed, which we would argue has important effects on their findings.

In summary, we here show suggestive evidence that the macroeconomic conditions of a country are reflected in its inhabitants’ brains. Our results suggest that human development is associated with larger brains with greater cortical surface and hippocampi, while inequality is most consistently associated with lower hippocampal volume and thinner global cortical thickness.

## Supporting information

10.1192/j.eurpsy.2025.10060.sm001 Medel et al. supplementary material Medel et al. supplementary material

## Data Availability

Group-level data and scripts can be requested from authors.

## Supplementary material

The supplementary material for this article can be found at http://doi.org/10.1192/j.eurpsy.2025.10060.
