# Supplementary material for: Human development, inequality, and their associations with brain structure across 29 countries
Source: Eur Psychiatry. 2025 Jul 16;68(1):e100. doi: 10.1192/j.eurpsy.2025.10060 (PMC12344469; doi:10.1192/j.eurpsy.2025.10060)
Supplement: Medel et al. supplementary material [file S0924933825100606sup001.docx]

***Supplementary Information for:***

**Human development, inequality, and their associations with brain structure across 29 countries**

V Medel, LM Alliende, RAI Bethlehem, J Seidlitz, GV Ringlein, C Arango, A Arnatkevičiūtė, L Asmal, M Bellgrove, V Benegal, M Bernardo, P Billeke, J Bosch-Bayard, R Bressan, GF Busatto, MN Castro, T Chaim-Avancini, M Costanzi, L Czepielewski, P Dazzan, C de la Fuente-Sandoval, CM Díaz-Caneja, AM Diaz-Zuluaga, S Du Plessis, FLS Duran, S Fittipaldi, A Fornito, NB Freimer, A Gadelha, CS Gama, R Garani, C Garcia-Rizo, C Gonzalez Campo, A Gonzalez-Valderrama, S Guinjoan, B Holla, A Ibañez, D Ivanovic, A Jackowski, P Leon-Ortiz, C Lochner, C López-Jaramillo, H Luckhoff, R Massuda, P McGuire, J Miyata, R Mizrahi, R Murray, A Ozerdem, PM Pan, M Parellada, L Phahladira, JP Ramirez-Mahaluf, R Reckziegel, TR Marques, F Reyes-Madrigal, A Roos, P Rosa, G Salum, F Scheffler, G Schumann, M Serpa, DJ Stein, A Tepper, J Tiego, T Ueno, J Undurraga, EA Undurraga, P Valdes-Sosa, I Valli, M Villarreal, TT Winton-Brown, N Yalin, F Zamorano, M Zanetti, for cVEDA, AM Winkler, S Evans-Lacko and NA Crossley.

**Content:**

1. Supplementary Figures
   1. Figure S1. Flowchart showing MRI sample selection.
   2. Figure S2. Changes across years in HDI and Inequality (adjusted) in included countries with more than one dataset acquired in different years.
   3. Figure S3. Effect of the use of 1.5T MRI scanners in the studies included in the meta-regression analyses.
   4. Figure S4. Sensitivity analyses using the year in which MRI images were likely obtained to model the level of development and inequality of the country.
2. Supplementary Tables
   1. Table S1. Characteristics of samples included.
   2. Table S2. HDI and Inequality (adjusted) of the countries included.

**Figure S1.**

***Figure S1. Flowchart showing MRI sample selection.***

**Figure S2.**

***Figure S2. Changes across years in HDI and Inequality (adjusted) in included countries with more than one dataset acquired in different years.*** *Note that imaging data were acquired in a relatively short range of time, particularly for the inequality data for which information was available. This restricted the possibility of performing analyses focusing on changes within countries.*

**Figure S3.**

***Figure S3. Effect of the use of 1.5T MRI scanners in the studies included in the meta-regression analyses.*** *Z-values are plotted. Using a lower magnetic field MRI machine did not have a significant effect on any of the main analyses reported in the manuscript.*

**Figure S4.**

**Figure S4. Sensitivity analyses using the year in which MRI images were likely obtained to model the level of development and inequality of the country.** Sensitivity analysis described as “Years” in green, comparing it to the main results of the paper (“Static” analysis). Inequality analyses are the most divergent. However, we were able to include 51% of the datasets due to lack of publications associated with them to inform acquisition date, or because no inequality index for that year and country was published by the United Nations. For the HDI analysis, this figure was 70%.

**Table S1.**

Please refer to Table_S1_R2.xlsx

**Table S2.**

| **Country** | **HDI** | **Inequality** |
| --- | --- | --- |
| Switzerland | 0.955 | 0.83378650 |
| Germany | 0.947 | 1.48770561 |
| Sweden | 0.945 | -0.02381461 |
| Australia | 0.944 | 1.32042528 |
| The Netherlands | 0.944 | 0.32042528 |
| Finland | 0.938 | -1.61413539 |
| United Kingdom | 0.932 | 0.65130395 |
| Belgium | 0.931 | 0.39554383 |
| Canada | 0.929 | 0.98402361 |
| United States of America | 0.926 | 4.51674328 |
| Austria | 0.922 | -0.90629717 |
| Israel | 0.919 | 2.92642250 |
| Japan | 0.919 | 0.12642250 |
| South Korea | 0.916 | 2.55914217 |
| Spain | 0.904 | 4.29002083 |
| France | 0.901 | -0.07725950 |
| Italy | 0.892 | 2.32089950 |
| Poland | 0.880 | -2.54822183 |
| Chile | 0.851 | 4.13473495 |
| Argentina | 0.845 | 1.10017428 |
| Russia | 0.824 | -3.27078805 |
| Turkey | 0.820 | 3.00617150 |
| Cuba | 0.783 | NA |
| Mexico | 0.779 | 5.02000695 |
| Colombia | 0.767 | 5.15088561 |
| Brazil | 0.765 | 7.83936539 |
| China | 0.761 | -1.08367505 |
| South Africa | 0.709 | 11.51679917 |
| India | 0.645 | 2.44815206 |

***Table S2. HDI and Inequality (adjusted) of the countries included.*** *Data obtained from the United Nations’ 2019 report. Inequality metric has been adjusted for leve lof development.*
